# Prevalence and correlates of low birth weight in India: findings from national family health survey 5

**DOI:** 10.1186/s12884-023-05726-y

**Published:** 2023-06-20

**Authors:** Damini Singh, Sayantani Manna, Manish Barik, Tanveer Rehman, Srikanta Kanungo, Sanghamitra Pati

**Affiliations:** Division of Public Health Research, ICMR-Regional Medical Research Centre, Bhubaneswar-23, Bhubaneswar, Odisha India

**Keywords:** NFHS-5, Low-birth-weight, Neurodevelopmental disorder, NICU, INAP, KMC, Neonatal and infant mortality

## Abstract

**Background:**

Childhood mortality and morbidity has become a major public health issue in low-middle-income countries. However, evidence suggested that Low birth weight(LBW) is one of the most important risk factors for childhood deaths and disability.This study is designed to estimate the prevalence of low birth weight (LBW) in India and to identify maternal correlates associated with LBW.

**Methods:**

Data has been taken from National Family Health Survey 5 (2019–2021) for analysis. 149,279 women belonging to reproductive age group (15–49) year who had last recent most delivery preceding the NFHS-5 survey.

**Results:**

Mother's age, female child, birth interval of less than 24 months, their low educational level, low wealth index, rural residence, lack of insurance coverage, women with low BMI, anaemia, and no ANC visits during pregnancy are predictors that contribute to LBW in India. After adjusting for covariates, smoking and alcohol consupmtion is strongly correlated with LBW.

**Conclusion:**

Mother’s age, educational attainment and socioeconomic status of living has a highly significant with LBW in India. However, consumption of tobacco and cigarrettes are also associated with LBW.

**Supplementary Information:**

The online version contains supplementary material available at 10.1186/s12884-023-05726-y.

## Background

The Global public health system continues to face significant obstacles, particularly in low-middle-income countries (LMICs), regarding maternal and child healthcare [1, 2]. One of the main objectives of the Sustainable development goals(SDGs) known as the health goal (goal No. 3) is to "ensure healthy lives and promote well-being for all at all ages," with family planning, information and education, and the inclusion of reproductive health into national strategies and programs being the key targets [3].Low birth weight has become the new public health threat at global level as it is one of the strongest risk factor associated with neonatal mortality and morbidity [4, 5]. LBW contributes 60–80% of neonatal deaths across the globe [6]. According to statistics, more than 20 million infants worldwide in 2015 weighed less than 2500 g at birth, making up at least 15%-20% of all children [7, 8]. The World Health Organization(WHO) has defined Low Birth Weight (LBW) as “a birth weight of less than 2,500 g at the time of birth, regardless of the gestational age” [9, 10]. The newborn must be weighed within the first hour of life before the physiological weight loss begins. Their birth weight determines how vulnerable they are to the risk of childhood illnesses and dying during their early childhood [11]. Preterm birth (28 to 37 weeks) or intrauterine growth restriction (babies that are tiny for gestational age and weigh less than the 10th percentile at mature) can also result in cases of LBW [12].Studies reflect that adolescent pregnancies have adverse effects on both mother and child health [13]. Infants born with LBW are more vulnerable and have higher chances of recurrent hospitalization, neurodevelopmental disorders, chronic morbidities, and under-5 moratalities [14–17]. Children with LBW are four-fold higher at risk of neonatal death when compared with their counterparts [18].

Studies have shown that LBW is the key predictor for neonate and infant mortality [19]They are more likely to develop congenintal heart anomalies and complications such as sepsis, respiratory distress syndrome and metabolic disturbances [20, 21]. Higher the growth impairment, the higher the risk of childhood death. Conditions such as insulin resistance, dyslipidemia, and high blood pressure are intensely related to LBW, resulting in increased rates of cardiovascular, metabolic and renal diseases and henceforth adult chronic diseases [22]. Research says that children with preterm births and LBW have resulted in developmental disabilities such as cerebral palsy, autism spectrum disorder and learning disability [23]. Also, they are likely to be poor in academic performance during their schooling years [14–17] Studies conducted in population-attributable-fractions in US populations have included an assessment of the Georgia Pregnancy Risk Assessment Monitoring System that has estimated 42% of Cerebral Palsy (CP) cases and 13% of ID cases were attributable to LBW, an assessment of Autism and Development Disabilities Monitoring Network that estimated 12% of ASD cases are attributable to Pre-term-birth (PTB), LBW and Caesarean delivery [23].

Advancements in medical technologies have enhanced the survival rates of infants with LBW. However, this have also increased the health care costs of bringing up these children [24]. Children with LBW are more susceptible to the length of stay(LOS) in hospitals, especially in the NICU. This creates a financial burden on the payer [25]. Research says that the LOS of the child in NICU depends on the ability of the payer to pay and this determines the discharge of the child [26]. Also, mothers of preterm births have a traumatic experience during LOS of the child in NICU [27]. This resulted in increased rates of stress and anxiety among those mothers as compared to mothers of full-term children [28]. They become emotionally vulnerable and the risk for psychological distress increases. Negative outcomes, such as childhood behavioral and emotional issues and neurodevelopmental delay, are in turn associated with psychological distress, which is characterized in this context as varied degrees of symptoms of depression, anxiety, and perinatal-specific post-traumatic stress [26]. Studies have shown that there is a strong association between maternal stress and LOS of children in the NICU [24].

A report published by UNICEF-WHO on LBW stated the prevalence as 26.4% in Southern Asia which was five times higher than Eastern Asia 5.1% in 2015. Member States of the World Health Assembly (WHA) 65th session employed the goal of a 30% worldwide decline in low birthweight between 2012 and 2025. Reporting on progress continues to be challenging, though. Since 2000, there has been no significant improvement in the rate of LBW babies, especially from 2010 to 2015. Without accelerated preventative measures, we will not be able to reduce LBW by 30% by 2025. Due to unavailability of data, the regional prevalence for India has not been evaluated in that report [29].

According to WHO (2004), prematurity and LBW account for 18.3 million disability-adjusted life years (DALYs) in the South-East Asian Region [30]. In order to measure population health, disability adjusted life years (DALYs), a summary metric, integrate mortality, morbidity, and disability [31]. In order to find accomplishments, unmet requirements, and possibly unanticipated rising risks to population health, this statistic explains the causes and predictors of mortality and morbidity. Studies have explained the ranks of the age-standardized DALYs and the shifts in ranks of various causes and discussed the comparative burden of communicable, maternal, neonatal and nutritional and non-communicable diseases.

### Rational for the study

There has not been much studies published on the prevalence of low birth weight in India and there is a dearth of national-level source for birth weight statistics, even though India has the greatest burden of LBW data are available either in birth certificate forms or in hospital discharge data forms. The NFHS of India, equivalent to the Demographic Health Survey (DHS), in its third round (NFHS-3, conducted in 2005–06) collected data on the birth weight of infants by the maternal recall, while asking mothers who had institutional deliveries to show their health cards, where the birth weight of the child is recorded. Similarly, during the NFHS round 5, the birth weight of the child has been recorded.

Several changes have taken place since 2005–06, thereby there is a need for a new study explaining on the current scenario of the country regarding LBW. There is also a need for studies investigating potential factors contributing to the high prevalence of LBW in India. There must be a portal for LBW as soon as the child is born, his weight should be recorded and he should be under supervision up to five years of age to prevent under-5 mortality. Findings from such studies can be used to develop interventions and policies focusing LBW in India.

### Objectives

#### Primary objectives

- 1. To estimate the prevalence of Low birth weight for institutional births in India

#### Secondary objectives

- 2. To identify the maternal correlates and their association with LBW.
- 3. To determine the association between lifestyle factors such as consumption of alcohol, tobacco, and cigarettes with LBW.

## Material and methods

### Source of information

This study is based on the data from the National Family Health Survey (NFHS) 5, a nationwide survey to scientifically investigate health and its social determinants and related economics in India. It gives information for 707 disticts, 28 states and 8 union territories. The nationwide data collection spanned from Phase I from 17 June 2019 to 30 January 2020 covering 17 states and 5 UTs and Phase-II from 2 January 2020 to April 2021 covering 11 states and 3 UTs [32].

### Study design

India is the world's second-most populous country (1.3 billion population), with 28 states and eight union territories (UTs). Each state and UT are further divided into districts. Districts are subdivided into census enumeration blocks and wards in urban areas and villages/taluks in rural areas. For this dissertation, data collected through the women individual schedules were used to create the dependent and independent variables. As the data had been collected as a part of an observational study, the study design for this research is also similar to a cross-sectional study.

### Study population

**Fig. 1:**
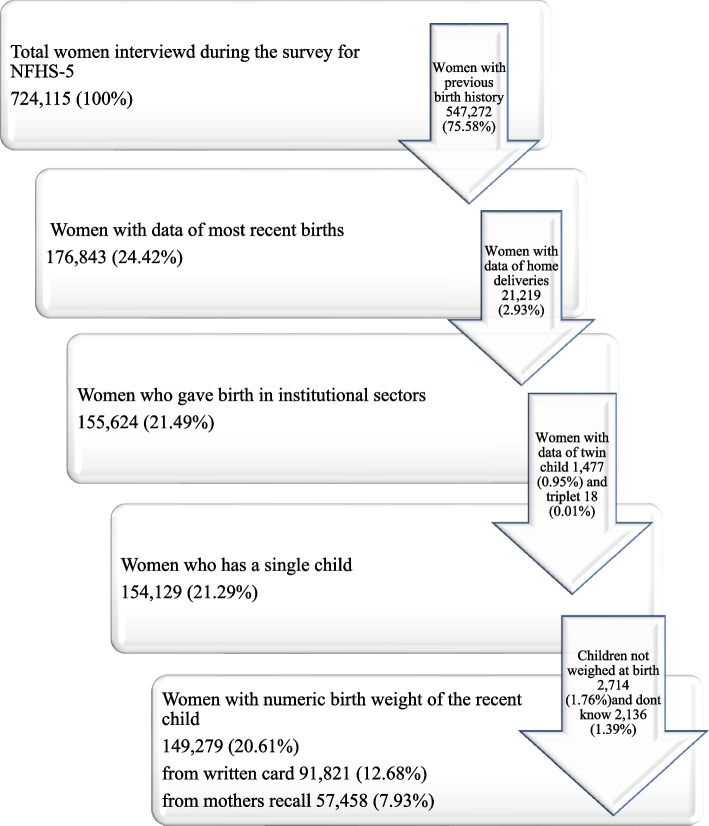
Selection criteria for the study population (unweighted)

NFHS 5 gathered information from 636,699 households, 724,115 women, and 101,839 men. Two stages of stratification were used to create the NFHS-5 sample. The sampling frame used to choose the PSUs(Population Sample Units) was the census from 2011. PSUs were Census Enumeration Blocks (CEBs) in urban regions and villages in rural areas. Less than 40-household PSUs were connected to the PSU that was closest to them. Villages were chosen from the sample frame inside each rural stratum with a probability proportionate to size (PPS). Prior to the main survey, a thorough household mapping and listing operation was carried out in each chosen rural and urban PSU. Selected PSUs with at least 300 estimated households were divided into pieces with 100 to 150 households each. The survey used systematic sampling with probability proportional to segment size, and two of the segments were randomly chosen. An NFHS-5 cluster is thus either a PSU or a PSU section. In the second step, 22 homes were randomly chosen using systematic sampling from each chosen rural and urban cluster. Volume II of the national report provides a thorough explanation of sample design, weight computation, standard error estimation, and techniques to improve data quality measurements. In this present study a total of 149,279 women aged 15–59 years were included for the analysis who were interviewed by individual schedule [33], the detailed sample derivation has been given in the Fig. 1.

#### Inclusion criteria-

• The most recent child born in the family, to minimize the possibility of change in several maternal correlates over time.
• Children delivered in healthcare institutions in India, to eliminate the imprecision of birth weight taken at home.
• Single born child, because multiple births such as twins, and triplets (more than one child in one delivery) have an influence on the birth weight of the children.

Birth weight data were collected from health cards or hospital discharge cards or mother’s self-reported data.

### Sampling technique

Villages and census enumeration blocks were chosen from districts in rural and urban areas, respectively, through a two-stage sampling procedure. Data collection was done using CAPI (Computer-assisted personal interview) from June 2019 to April 2021 with an inbuilt schedule and proper maintenance of confidentiality of respondents’ answers. NFHS-5 methodology, including selecting households and data collection procedures, has been meticulously described and published [32].

### Study procedure and sample size

A total number of women participants in the entire study design is 724115 out of which only 176843 (24.42%) participants had most recent birth history preceding the survey. Participants who delivered in healthcare facilities 155624 (21.49%) were considered for further analysis. Women who delivered single child 154129 (21.29%) with evidence of numeric birth weight 149279 (20.61%) was the confirmed study population.

Women with previous child birth history 547272 (75.58%), home deliveries 21219 (2.93%), with data of twins 1477(0.95%) and triplets 18 (0.01%) and child not weighted at birth 2714 (1.76%) or don’t know the exact weight 2136 (1.39%) were dropped from the study Fig. 1.

### Dependent variable

Children were classified to have LBW if their birth weight was less than 2500 g.

### Independent variable

**Fig. 2:**
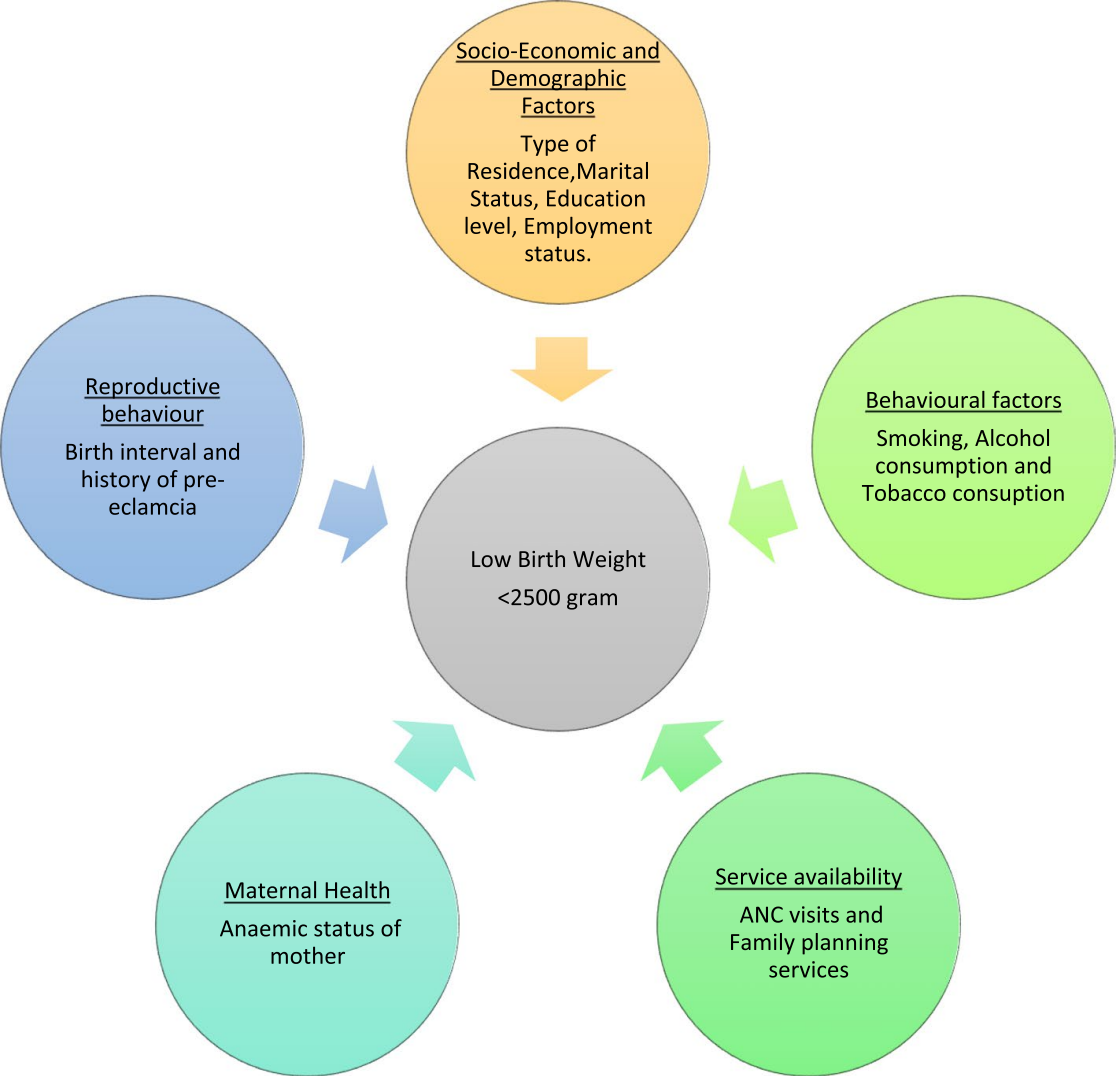
Conceptual framework for low birthweight

Among the sociodemographic characteristics of mother or family are the mother's age, level of education, wealth index, marital status, religious background, and location of residence. Age of mother, birth order, birth interval, complications during pregnancy, and health behaviours such as smoking and alcohol use were among the reproductive characteristics of the mother. These variables were taken into analysis from the conceptual framework Fig. 2

ANC status comprised the initial ANC visit's timing, the total number of ANC visits, the participant's pregnant tetanus shot, the location of the birth, and service accessibility. ANC status has been divided into three sub-categories namely no ANC visits, ≤ 4 ANC visits and > 4 ANC visits [34].

Among the anthropometric measurements are the mother's body mass index was categorized as “Underweight” (less than 18.5 kg/m^2^), “Normal” (18.5– 24.9 kg/m^2^), “Overweight” (25.0–29.9 kg/m^2^), and “Obese” (≥ 30.0 kg/m^2^) [35]. Anemia was classified as blood hemoglobin level < 12.0 g/ dL, for nonpregnant women aged 15–49 years, which was further categorized as “mild” (11.0–11.9 g/dL), “moderate” (8.0–10.9 g/dL), and “severe anemia” (< 8.0 g/dL); any anemia was defined as blood hemoglobin level < 11 g/dL,for pregnant women and further categorized as “mild” (10.0–10.9 g/dL), “moderate” (7.0–9.9 g/dL), and “severe anemia” (< 7.0 g/dL) [36].

### Data analysis

STATA 16.0 was used to clean and analyse the data (Stata Corp., Texas). For a continuous variable like age, we determined the mean and standard deviation. Additionally, we looked at weighted profiles in both sexes and reported them as percentages and figures [32]. The frequency (n, n%) and p-value of the prevalence of low birth weight among other categorical factors (such as age group, gender, area of residence, educational attainment, life partner, caste, employment status, national region, and wealth index) were shown. A p-value of 0.05 or below was considered statistically significant. We used multivariate logistic regression analysis to further evaluate the statistically relevant factors. The adjusted odds ratio (AOR) with a 95% confidence interval (CI) was used to express the weighted association from regression analysis.

### Ethical consideration

This study is based on secondary data obtained from NFHS 5 (2019–21) with no personal identifiers and hence there is no participant risk. The data were requested from International Institute of Population Sciences (IIPS), Mumbai through proper channel and appropriate permission was taken. The same have been properly acknowledged and referenced wherever required.

As a result of literature review, we have identified potential confounders of LBW and its association among different independent variables. We have found that these confounders are also common risk factors of LBW providing were selected and adjusted for it. Variables include place of residence, marital status, birth interval, birth order and alcohol consumption.

## Results

After applying the inclusion criteria, the number of participants involved were 149,279 within the age range from 15 to 49 years (Table 1). The mean age of the participants was 27.02 and (± 4.93) years. The majority of the study population belonged to 25–34 years age-group (58.43%), in rural setup (70.19%). However, nearly half of the population was from OBC category (45.62%) and had completed their secondary level of education (53.87%). The Hindu female (80.33%) and women with normal BMI (60.49%) were large in number.

**Table 1:** Socio-demographic characteristics of study population

Characteristics		Weighted (n, %)
**Age group (*n* = 149,279)**	15–24 years	49215, 33
	25–34 years	87219, 58.4
	≥ 35 years	12844, 8.6
**Residence (*n* = 149,279)**	Urban	44505, 29.8
	Rural	104773, 70.2
**Caste (*n* = 141,872)**	Scheduled Caste	33184, 23.4
	Scheduled Tribe	13799, 9.7
	Other Backward Class	64728, 45.6
	None of the caste	28910, 21.3
	Not sure of their caste	1250, 1
**Educational attainment (*n* = 149,279)**	No formal education	23875, 15.9
	Completed primary education	16459, 11.1
	Completed secondary education	80410, 53.8
	Higher secondary and above	28535, 19.2
**Body Mass Index (*n* = 145,385)**	Underweight	26136, 18
	Normal	87944, 60.5
	Overweight	23770, 16.35
	Obese	7535, 5.2
**Religion (*n* = 149,279)**	Hindu	119923, 80.3
	Muslim	22603, 15.8
	Christian	3037, 2.04
	Other religions	3715, 2.49
**Employment (*n* = 22,764)**	Currently unemployed	17862, 78.5
	Currently employed	4902, 21.5
**Life Partner (*n* = 149,279)**	Lives without partner	1746, 1.2
	Lives with partner	147279, 98.8
**Wealth quintile (*n* = 149,279)**	Poorest quintile	28686, 19.2
	Poorer quintile	30794, 20.6
	Middle quintile	30496, 20.4
	Richer quintile	30833, 20.6
	Richest quintile	28469, 19.2
**Pregnancy Complications (*n* = 142,814)**	Absent	31084, 21.8
	Present	111730, 78.2
**Health Insurance Coverage (*n* = 149,279)**	Absent	112228, 75.1
	Present	37051, 24.9

Supplementary: 1 represents the prevalence of LBW infants among the most recent deliveries in healthcare facilities in India by states and its union territories. Nearly, one fifth (17.06%) of the infants had LBW. States like Punjab (21.36%) and Delhi (20.11) had the highest prevalence of LBW followed by Madhya Pradesh (19.47%), Uttar Pradesh (19.20%) and Daman Diu and Dadar and Nagar Haveli (19.07%). Whereas states like Nagaland (3.38%) and Mizoram (3.36%) had the lowest prevalence of LBW.

**Fig. 3:**
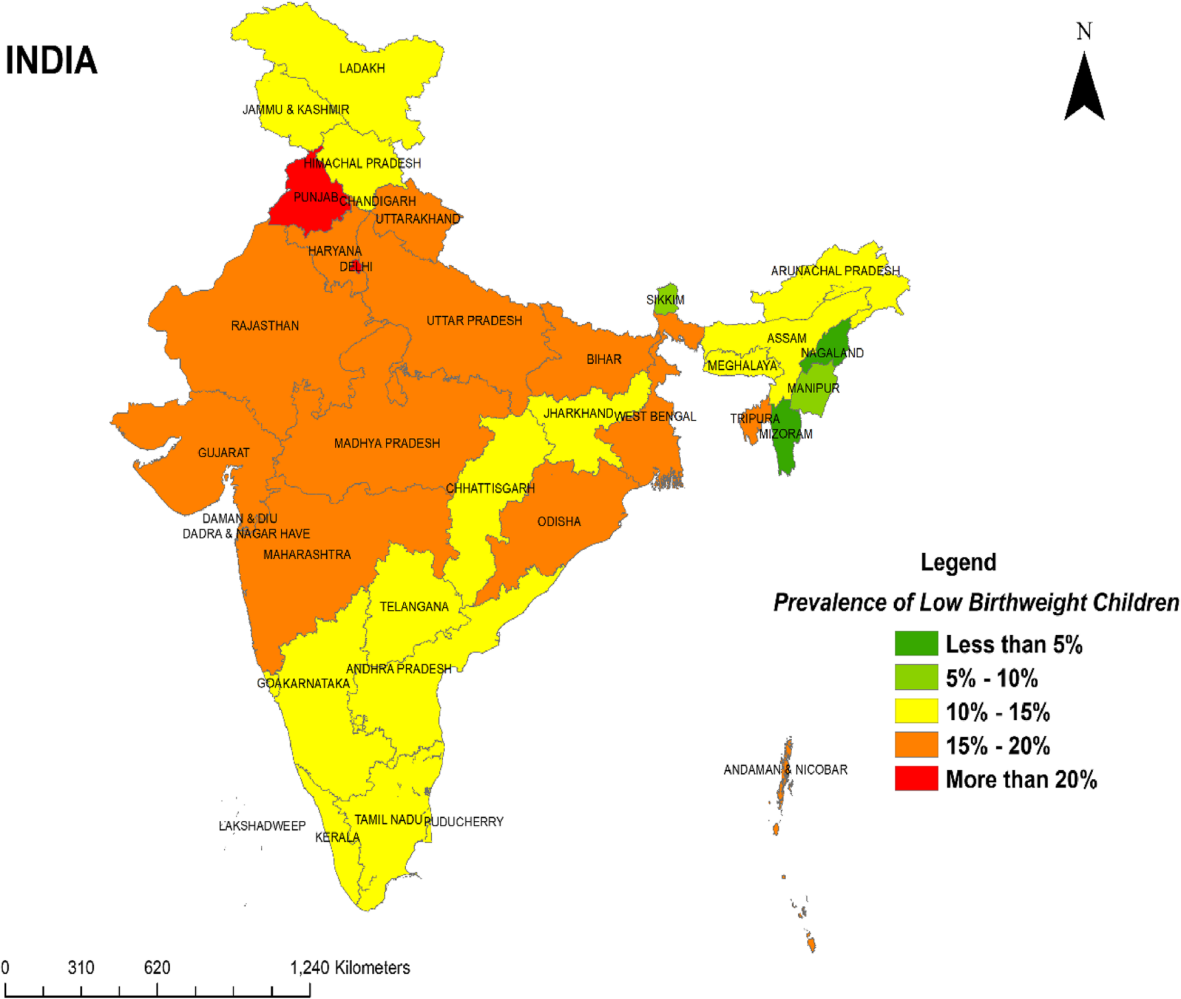
Classification of states as per the number of low-birth-weight child deliveries in healthcare facilities

Figure 3 represents the prevalence of low-birth-weight across Indian states and Union territories. Low prevalence refers to the n% distribution across states i.e., < 5%, 5%-10%, 10%-15%, 15%-20% and > 20%.

Table 2 represents the prevalence of LBW among different correlates of the mother. Variables such as the age of the mother, gender of the child, birth order, birth interval in months, educational attainment, wealth quintile, caste, place of residence, BMI status, anaemia status, number of ANC visits, iron supplementations and tetanus injection during pregnancy were significantly associated with LBW of infants (*P* < 0.05). Correlates such as pregnancy complications, caesarean mode of delivery, employment status, and marital status were not significantly associated with our outcome variable. The findings of the chi-square confirmed disparities between the two groups, which were distinguished by the presence or absence of LBW, in terms of the mother's socioeconomic status, educational attainment and caste group.

**Table 2:** Prevalence of low birth weight in selected maternal correlates among the most recent child delivered in healthcare facilities, NFHS -5

Characteristics		Birth Weight n, %, (95% CI)		*P*-value	Chi-square
	**Categories**	< 2500 g	≥ 2500 g		
**Age (in years)**	15–24	9396, 19.1, (18.7–19.4)	39819, 80.9, (80.55–81.25)	< 0.001	252.10
	25–34	14029, 16.1, (15.8–16.3)	73189, 83.9, (83.6–84.5)		
	35 and above	2047, 15.9, (15.3–16.5)	10797, 84.1, (83.4–84.6)		
**Residence**	Urban	7166, 16.1, (15.76–16.44)	37339, 83.9, (83.55–84.24)	< 0.001	25.31
	Rural	18307, 17.4, (17.4–17.7)	86466, 82.6, (82.2–82.7)		
**Caste**	Schedule Caste	6191, 18.6, (18.3–19.1)	26993, 81.4, (80.9–81.7)	< 0.001	216.17
	Schedule Tribe	2457, 17.8, (17.1–18.4)	11342, 82.2, (81.5–82.8)		
	OBC	10732, 16.6, (16.3–16.8)	53995, 83.4, (83.1–83.7)		
	None of them	4628, 16.1, (15.5–16.4)	24282, 83.9, (83.5–84.4)		
	Don’t know	283, 22.6, (20.3–25.1)	967, 77.4, (74.9–79.6)		
**Education**	No Education	4625, 19.3, (18.8–19.8)	19249, 80.7, (80.1–81.1)	< 0.001	401.66
	Primary	3313, 20.3 (19.5–20.7)	13146, 79.7, (79.2–80.4)		
	Secondary	13768, 17.1, (16.8–17.3)	66641, 82.9, (82.6–83.1)		
	Higher	3767, 13.2, (12.8–13.9)	24767, 86.8, (86.9–87.9)		
**Wealth quintile**	Poorest	5826, 20.3, (19.4–20.8)	22861, 79.7, (79.2–80.1)	< 0.001	361.58
	Poorer	5787, 18.8, (18.3–19.2)	25007, 81.2, (80.7–81.6)		
	Middle	5038, 16.5 (16.1–16.9)	25457, 83.5, (83.1–83.8)		
	Richer	4873, 15.8, (15.4–16.2)	25960, 84.2, (83.7–84.6)		
	Richest	3950, 13.8, (13.4–14.2)	24519, 86.2, (85.7–86.5)		
**Health Insurance Coverage**	No	19776, 17.6, (17.4–17.8)	92452, 82.4, (82.1–82.6)	< 0.001	78.85
	Yes	5697, 15.4, (15.1–15.7)	31354, 84.6, (84.2–84.9)		
**Body Mass Index**	Underweight	5586, 21.4, (20.8–21.9)	20549, 78.6, (78.1–79.1)	< 0.001	569.65
	Normal	14690, 16.7, (16.4–16.9)	73254, 83.3, (83.05–8.5)		
	Overweight	3442, 14.5, (14.3–14.9)	20327, 85.5, (85.1–85.9)		
	Obese	1090, 14.4, (13.6–15.2)	6445, 85.6, (84.7–86.3)		
**ANC Visits**	No	1375, 20.3, (19.3–21.3)	5384, 79.7, (78.7–80.6)	< 0.001	126.88
	4 visits	12522, 17.7, (17.4–18.1)	58148, 82.3, (81.9–82.5)		
	< 4 visits	11575, 16.1, (15.8–16.3)	60274, 83.9, (83.2–84.1)		
**Anaemia**	Severe	615, 21.1, (19.9–22.8)	2305, 78.9, (77.3–80.3)	< 0.001	74.57
	Moderate	7538, 17.5, (17.1–17.8)	35490, 82.5, (82.1–82.8)		
	Mild	6503, 17.1, (16.7–17.4)	31498, 82.9, (82.5–83.2)		
	Not Anaemic	9877, 16.5, (16.2–16.8)	49896, 83.5, (83.1–83.7)		
**Marital Status**	Without Partner	338, 19.3, (17.3–21.2)	1408, 80.7, (78.7–82.4)	0.349	0.88
	With Partner	25135, 17.1, (16.8–17.2)	122397, 82.9, (82.7–83.1)		
**Employment**	Not Working	3072, 17.2, (16.6–17.7)	14789, 82.8, (82.2–83.3)	0.599	0.27
	Currently Working	872, 17.8, (16.7–18.8)	4029, 82.2, (81.1–83.2)		
**Birth Order**	First	10095, 18.2, (17.9–18.5)	45152, 81.8, (81.4–82.1)	0.001	102.32
	Second & third	12731, 16.2, (15.9–16.4)	66013, 83.8, (83.5–84.1)		
	Four	2647, 17.3, (16.7–17.9)	12641, 82.7, (82.0–83.2)		
**Birth Interval**	< 24 months	4431, 17.5, (16.9–17.9)	20940, 82.5, (82.1–83.01)	0.001	48.92
	> 24 months	11030, 15.9, (15.6–16.2)	58138, 84.1, (83.7–84.3)		
**Sex of Child**	Male	12660, 15.7, (15.4–15.9)	67936, 84.3, (84.1–84.5)	0.001	159.85
	Female	12813, 18.6, (18.3–18.9)	55869, 81.4, (81.1–81.6)		
**Pregnancy Complications**	No	5294, 17.1, (16.6–17.4)	25789, 82.9, (82.4–83.3)	0.768	0.08
	Yes	18853, 16.9, (16.6–17.1)	92877, 83.1, (82.9–83.3)		
**Caesarean section**	No	18706, 17.1, (16.8–17.3)	90755, 82.9, (82.6–83.1)	0.303	1.06
	Yes	6766, 16.9, (16.2–17.6)	33051, 83.1, (82.6–83.3)		
**Iron Supplementation**	No	2864, 18.9, (18.2–19.5)	12286, 81.1, (80.4–81.7)	> 0.001	64.93
	Yes	22525, 16.8, (16.6–17.1)	111236, 83.2, (08.2–08.5)		
**Tetanus Injection**	No	1198, 20.1, (19.1–21.1)	4763, 79.9, (78.8–80.9)	> 0.001	33.23
	Yes	24076, 16.9, (16.7–17.1)	118409, 83.1, (82.9–83.2)		
**Doesn’t use Cigarette and Tobacco**	No	851, 20.29, (19.09–21.55)	3342, 79.71, (78.45–80.91)	0.012	6.31
	Yes	24622, 16.9, (16.8–17.1)	120463, 83.1, (82.8–83.2)		
**Do you drink Alcohol**	No	25341, 17.6, (16.6–17.5)	123232, 82.9, (82.7–83.1)	< 0.001	19.56
	Yes	132, 18.7, (15.8–21.7)	573, 81.3, (78.8–83.9)		
**Frequency of Alcohol Consumption**	Everyday	90, 28.39, (23.41–33.59)	228, 71.61, (66.40–76.58)	< 0.001	21.53
	Once a week	156, 22.5, (19.3–25.7)	539, 77.5, (74.2–80.6)		
	Less than once a week	143, 13.5, (11.5–15.5)	917, 86.5, (84.3–88.5)		

Among the infants with LBW, women of 15 to 24 years had the highest prevalence of LBW 19.09% (95%CI: 18.74–19.44). Women with higher levels of education (13.20%) had a lower prevalence of LBW as compared to women with no primary education (19.37%). Scheduled caste (18.66%) and scheduled tribe (17.81%) women were considered to be vulnerable to LBW infants. Underweight mothers (21.37%) had higher prevalence for LBW as compared to obese (14.48%) and normal BMI (16.70%) of the mother. Women with pregnancy complications had a 16.87% of LBW prevalence. Mothers with no ANC visits (18.90%), no iron supplementations (18.90%), and no tetanus injections during pregnancy (20.10%) had the highest prevalence among any other variables. However, modifiable lifestyle habits such as consuming cigarettes, tobacco (20.29%) and alcohol (18.77%) are other risk factors associated with LBW.

Supplementary 2 and Table 3 summarize the results of univariate and multivariate logistic regression of various factors associated with LBW among single child born in healthcare facilities of India, based on NFHS-5 (2019–2021). There were twenty-one independent variables, both continuous and categorical (characteristics of interest) out of which sixteen independent variables were significantly associated with LBW.

**Table 3:** Multivariate logistic regression analysis depicting associations of LBW with various socio-demographic attributes

Characteristics	Adjusted Odds Ratio	95% Confidence Interval		*P* value
		Lower	Upper	
**Age of mother**				
15–24 years	1.09	0.99	1.19	0.084
25–34 years	1.01	0.94	1.08	0.249
≥ 35 years	Ref			
**Residence**				
Urban	Ref			0.002
Rural	0.89	0.83	0.96	
**Education**				
No formal education	1.45	1.31	1.61	< 0.001
Completed primary education	1.58	1.42	1.76	
Completed secondary education	1.31	1.19	1.43	
Higher secondary and above	Ref			
**BMI**				
Underweight	1.35	1.19	1.55	< 0.001
Normal	1.11	0.98	1.25	0.109
Overweight	1.02	0.90	1.17	0.720
Obese	Ref			
**Wealth Quintile**				
Poorest	1.22	1.10	1.35	< 0.001
Poorer	1.14	1.03	1.25	0.010
Middle	1.07	0.97	1.18	0.176
Richer	1.05	0.95	1.15	0.346
Richest	Ref			
**Anaemia status**				
Severe	1.27	1.09	1.46	0.002
Moderate	1.01	0.94	1.06	0.957
Mild	1.01	0.95	1.08	0.660
Not anaemic	Ref			
**Sex of the Child**				
Female	1.28	1.22	1.35	< 0.001
Male	Ref			
**Birth Interval**				
< 24 months	1.07	1.01	1.14	< 0.001
> 24 months	Ref			
**Insurance Coverage**				
Yes	Ref			
No	1.18	1.12	1.25	< 0.001
**ANC Visits**				
No visits	1.19	1.08	1.33	< 0.001
< 4	1.04	0.99	1.09	0.155
> 4	Ref			

The univariate regression shows that the age of the mother, gender of the child, place of residence, insurance coverage, BMI status of the mother, wealth index, educational attainment, number of ANC visits, birth order, birth interval, caste, anaemia status, iron supplementation and tetanus injections during pregnancy were significantly associated with LBW (Table 4).

**Table 4:** Association between tobacco and alcohol consumption with low birth weight among most recent child delivered in healthcare facilities of India (NFHS-5)

Characteristics	Adjusted Odds Ratio	Confidence Interval		*p*-value
		Lower	Upper	
**Tobacco usage**				
No	Ref			
	1.24	1.12	1.37	< 0.001
**Alcohol consumption**				
Yes	1.08	0.87	1.35	0.473
No	Ref			

Adjusted for age, residence, caste and health insurance

Modifiable lifestyle habits such as the consumption of cigarettes and tobacco and the frequency of alcohol consumption were significantly associated with LBW. Whereas consumption of alcohol (p-value 0.3) was not significantly associated with LBW. However, mother having history of daily alcohol intake had greater chances of delivering LBW infants as compared to occasional drinkers and non-drinkers.

Table 3 shows that women with primary education had higher risk of delivering LBW infant (AOR:1.58 (95% CI: 1.42–1.76)) followed by mothers with no formal education (AOR:1.45 (95% CI: 1.31–1.61)). Mothers belonging to poorest (AOR: 1.22(95% CI: 1.10–1.35)) and poorer (AOR:1.14 (95% CI:1.03–1.25)) wealth quintile had higher odds of delivering LBW child with reference to mothers belonging to richest quintile. Underweight mothers (AOR: 1.35(95% CI:1.19–1.55)) were more likely to have LBW infants keeping obese mothers as reference. With the increase in severity of anaemia, the risk of having LBW infant also increases(AOR: 1.27(95% CI: 1.09–1.46)). As compared to male children, female children (AOR: 1.28 (95% CI: 1.22–1.35) had higher risk of having LBW. Mothers with history of no ANC visits during their last pregnancy (AOR: 1.19(95% CI: 1.08–1.33) had higher chances of having LBW babies.

Table 4 estimates the multivariable logistic regression with LBW as the outcome and cigarettes, tobacco and alcohol consumption as the exposure of interest. Here the outcome variable and exposure variable were adjusted for age of the mother, place of residence, caste group, and health insurance coverage. The adjusted multi- variable model revealed that women using cigarettes and tobacco had a higher risk of delivering low birth weight child (AOR:1.24 (95% CI: 1.12–1.37)) than those who were not using cigarettes and tobacco. It was significantly associated with LBW for the recent most child deliveries (*p*-value < 0.01), whereas LBW was not significant for women with history of alcohol consumption(p-value 0.473).

## Discussion

The aim of the study was to identify potential predictors of low birth weight (LBW) in India, and specifically to investigate how maternal age associated with the likelihood of LBW. The study found that several variables were potentially associated with LBW, including maternal age, the sex of the child, maternal education level, wealth index, religion, insurance coverage, location of residence, maternal body mass index (BMI), anemia status, history of stillbirths, birth spacing, and adequate antenatal care (ANC) visits. The majority of these variables, such as maternal age, education, insurance coverage, BMI, anemia, appropriate birth intervals, and sufficient ANC visits, were identified as modifiable factors that could potentially be improved in order to reduce the risk of LBW.**.**

The results of this study support other studies that show female new-borns have a greater risk of LBW than male neonates. Studies confirmed that this might be caused by female foetuses having greater levels of maternal glucose intolerance, which may have an influence on their birth weight [37, 38].

According to studies, singleton pregnancy women (women giving birth for the first time) were more susceptible than multiparous women (women who have given birth previously) with inadequate birth gaps to have a kid who is LBW. The study's results were consistent with this hypothesis. Further research revealed that moms with birth intervals of less than two years were most likely to produce LBW children than mothers with birth intervals of two or more years. These results supported those of earlier investigations, as well [39–41].

Based on the study's findings, mothers with higher educational attainment were less likely to deliver an LBW baby than uneducated mothers. We noticed a dose–response relationship between maternal education level and the likelihood of delivering an LBW infant i.e., the risk of having LBW child decreases with an increase in educational level of mothers. This was in line with the outcomes of other research conducted in India utilizing the NFHS-3 data [5, 30]. In accordance with the findings, babies born to mothers from lower income families (belonging to poorest and poorer wealth quintile) were more likely than babies born to mothers from higher-income families to be born LBW. Women with no education and/or understanding were likely to adapt in unhealthy behaviours (such as smoking, using drugs, tobacco or alcohol, etc.). Additionally, they could not afford access to necessary healthcare resources (such as antenatal care, tetanus injections or iron supplements), which would likely have had an impact on foetal growth. Interventions to raise the educational level of women and young girls were therefore crucial to reducing the prevalence of LBW in India [5]. These results coincided with those of previous studies [5, 42].

Insurance coverage had a crucial role in LBW in India. The National Health Mission (NHM) in India has been working for more than 15 years with the goals of expanding service coverage, maximising equity in the health sectors, and improving health outcomes, while focusing solely on reducing out-of-pocket expenditure (OOPE) and catastrophic health spending (CHS), particularly among the most disadvantaged, deprived, and vulnerable groups. However, no such insurance policies were available that can uptake the financial hardship for LBW child or sick child [43, 44]. Compared to mothers without insurance, those who gave birth to LBW children had a lower likelihood of doing so. Given the conflicting findings of earlier investigations, this was a novel finding of the study. Women with insurance coverage were less likely to deliver an LBW child as compared to those without health insurance. Research done in Arizona revealed that the lack of insurance was significantly associated with increased chances of having an LBW infant, in contrast to a study conducted in Cambodia that found no association between insurance coverage and LBW [41, 45].

Rural mothers had a protective factor of having an LBW baby, which was different from NFHS-3 findings. This outcome might be attributed to better dietary habits and novel prenatal care procedures [46–48].

Low micronutrient intake was associated with a low BMI. Foetal development may be hampered by pregnant mothers having low nutritional levels. According to research by Ramana and colleagues, consuming 30% to 50% more protein overall might lower the risk of having a baby that is underweight. Increased low birth weight and infant death were a result of the mother's low BMI. Low birth weight had been more common in moms who were underweight. According to the research, birth weight among the various castes had been associated to rising BMI and maternal weight growth during pregnancy. As per the World Health Organization and United Nations Children's Fund,96% of LBW births, are caused by low socioeconomic situations, poor diet, infections, and physical labour during pregnancy [49–52].

Evidences has shown that inadequate antenatal care (ANC) visits can have significant implications for the course of pregnancy and the health of new born. Studies have also found a strong association between LBW and inadequate ANC visits during pregnancy, which increases the likelihood of having a baby with LBW [53–55]. Notably, the association of mother's education with ANC and delivery care weakened in the NFHS-4 compared to the NFHS-3. After the NRHM was introduced in 2005, there may have been a sharp rise in institutional delivery and ANC care in NFHS-4. Additionally, a major increase in ANC quality over the past ten years may be attributable to a number of NRHM-related programmes, such as the Janani Suraksha Yojana (Safe Motherhood Scheme). Institutional delivery became the societal norm as a result of this growth in maternal health service use, which was especially pronounced among underprivileged groups (IIPS and ICF, 2017) [56].

Less than 5% of the mothers in the current research used cigarettes, compared to more than 50% of the fathers. There is dearth of evidence to suggest that smoking during pregnancy is statistically significantly associated with an increased odds ratio of delivering a low birth weight (LBW) baby. However, Kramer discovered that indoor smoke, cigarette smoking, and tobacco chewing were potentially significant and that their causal influence was proven in his meta-analysis [57]. Based on study findings, consumption of smoking and tobacco were significantly associated with LBW. However, numerous research had revealed a statistically significant association between LBW and cigarette use [58–60]. A Taiwan Birth Cohort Study demonstrates a strong association between maternal smoking and LBW and premature birth. 57.3% of the LBW babies in this research were born preterm, and 44% were small for gestational age(SGA). Birth weight was adversely affected by preterm delivery, and intrauterine growth restriction (IUGR) also increases low birth weight (LBW). In utero development and birth weight may be impacted by maternal smoking via the following potential processes. The concentration of nicotine, the key tobacco ingredient, is 15% greater in the placenta than it is in the mother's blood. Nicotine stimulates the release of maternal catecholamines, which constricts the uterus. Additionally, maternal smoking raises the amounts of carboxyhaemoglobin in the umbilical arteries, which causes hypoxia in the featus. Smoking by mothers may have an impact on LBW by lowering leptin levels [61–64]. The final trimester of pregnancy had the greatest impact on the birth weight of the child, particularly for mothers who smoke heavily (more than 8–10 cigarettes per day) [64–66].

### Strength and limitations

This study utilized a representative sample of women in India, obtained from nationally representative data, which allowed us to conduct an analysis that is representative of the country as a whole, and to provide a comprehensive understanding of LBW. Recall bias and reporting errors might be associated particularly with the LBW, and other variables, such as age, education level etc.

### Conclusion and recommendation

The determinants of LBW in India were evaluated in this study. The study's findings on LBW predictors can be utilised to both pinpoint high-risk individuals and forecast LBW trends. Further research is required to assess the possible causative impact of various indicators detected during pregnancy, such as BMI, anaemia, smoking, alcohol use, history of LBW, and others. The relationship between mothers age and LBW among new born in India was also examined in this study. According to the study findings, adolescent mothers are more likely to deliver children who have LBW. This evidence can inform intervention strategies for healthcare workers, providers, NGOs, policymakers, and public health professionals in India. In addition to that, there is a need of setting up a portal for LBW as soon as the children is born, their weight should be recorded and should be under supervision up to five years of age to prevent under-5 mortality. Similarly, the home-based neonatal care set-ups should be different for LBW child and child with normal weight. Awareness campaign and behaviour change communication strategies need to be revised for LBW child.

## Supplementary Information

**Additional file 1:** **Supplementary 1.** Distribution of low birth weight among Indian states and its Union Territories of the recent child born in healthcare facilities (NFHS-5). **Supplementary 2.** Univariate Logistic Regression of maternal correlates with LBW <2500grams as outcome variable among the recent most child delivered in healthcare facilities of India.

## Data Availability

All data are publicly available and can be accessed through The DHS Program, https://dhsprogram.com/data/.
