# Supplementary material for: Prevalence and correlates of low birth weight in India: findings from national family health survey 5
Source: BMC Pregnancy Childbirth. 2023 Jun 20;23:456. doi: 10.1186/s12884-023-05726-y (PMC10283257; doi:10.1186/s12884-023-05726-y)
Supplement: Supplementary file 1 — Additional file 1: Supplementary 1. Distribution of low birth weight among Indian states and its Union Territories of the recent child born in healthcare facilities (NFHS-5). Supplementary 2. Univariate Logistic Regression of maternal correlates with LBW <2500grams as outcome variable among the recent most child delivered in healthcare facilities of India. [file 12884_2023_5726_MOESM1_ESM.docx]

**Supplementary 1 : Distribution of low birth weight among Indian states and its Union Territories of the recent child born in healthcare facilities (NFHS-5)**

| **States and UTs** | **Birth Weight** | | **Total**  **n, %** |
| --- | --- | --- | --- |
|  | **< 2500grams**  **n, %** | **≥2500grams**  **n, %** |  |
| Andaman and Nicobar Island | 6, 16.57 | 28, 83.43 | 34, 0.02 |
| Andra Pradesh | 757, 14.81 | 4355, 85.19 | 5112, 3.42 |
| Arunachal Pradesh | 11, 10.06 | 98, 89.94 | 109, 0.07 |
| Assam | 620, 14.80 | 3567, 85.20 | 4187, 2.80 |
| Bihar | 2288, 16.16 | 11871, 83.84 | 14159, 9.48 |
| Chandigarh | 18, 16.39 | 95, 83.61 | 113, 0.08 |
| Chhattisgarh | 467, 14.26 | 2808, 85.74 | 3275, 2.19 |
| Daman & Diu & Dadra & Nagar Haveli | 12, 19.07 | 50, 80.93 | 62, 0.04 |
| Delhi | 419, 20.11 | 1665, 79.89 | 2084, 1.40 |
| Goa | 21, 12.62 | 149, 87.38 | 170, 0.11 |
| Gujarat | 1152, 16.68 | 5758, 83.32 | 6910, 2.19 |
| Haryana | 587, 19.36 | 2447, 80.64 | 3034, 2.03 |
| Himachal Pradesh | 102, 14.87 | 583, 85.13 | 685, 0.46 |
| Jammu & Kashmir | 120, 10.10 | 1066, 89.90 | 1186, 0.79 |
| Jharkhand | 527, 14.01 | 3234, 85.99 | 3760, 2.52 |
| Karnataka | 1054, 14.34 | 6294, 85.66 | 7348, 4.92 |
| Kerala | 541, 14.36 | 3230, 85.64 | 3771, 2.53 |
| Ladakh | 2, 10.69 | 19, 89.31 | 21, 0.01 |
| Lakshadweep | 1, 9.59 | 8, 90.41 | 9, 0.01 |
| Madhya Pradesh | 1712, 19.47 | 7078, 80.53 | 8790, 5.89 |
| Maharashtra | 2570, 18.93 | 11002, 81.07 | 13572, 9.09 |
| Manipur | 22, 7.40 | 272, 92.60 | 294, 0.20 |
| Mizoram | 4, 3.63 | 113, 96.37 | 117, 0.08 |
| Meghalaya | 45, 12.03 | 327, 87.97 | 372, 0.25 |
| Nagaland | 2, 3.68 | 66, 96.32 | 68, 0.05 |
| Odisha | 965, 17.55 | 4533, 82.45 | 5498, 3.68 |
| Puducherry | 13, 11.88 | 101, 88.12 | 114, 0.08 |
| Punjab | 632, 21.36 | 2327, 78.64 | 2959, 1.98 |
| Rajasthan | 1688, 16.76 | 8382, 83.24 | 10070, 6.75 |
| Sikkim | 4, 8.24 | 46, 91.76 | 50, 0.03 |
| Tamil Nadu | 1246, 14.93 | 7101, 85.07 | 8347, 5.59 |
| Telangana | 436, 12.15 | 3154, 87.85 | 3590, 2.41 |
| Tripura | 89, 18.82 | 383, 81.18 | 472, 0.32 |
| Uttar Pradesh | 4887, 19.20 | 20571, 80.80 | 25458, 17.05 |
| Uttarakhand | 186, 16.30 | 957, 83.70 | 1143, 0.77 |
| West Bengal | 2266, 18.37 | 10065, 81.63 | 12331, 8.26 |
| **Total**  **n, %** | 25473, 17.06 | 123806, 82.94 | 149279, 100.00 |

**Supplementary 2 : Univariate Logistic Regression of maternal correlates with LBW <2500grams as outcome variable among the recent most child delivered in healthcare facilities of India**

| **Socio-demographic haracteristics** | **Odds Ratio** | **95% Confidence Interval** | | **P value** |
| --- | --- | --- | --- | --- |
|  | | Lower | Upper |  |
| **Age of mother** | | | | |
| 15-24 years | 1.24 | 1.16 | 1.33 | <0.001 |
| 25-34 years | 1.01 | 0.94 | 1.08 |  |
| ≥35 years | Ref | | |  |
| **Residence** | | | | |
| Urban | Ref | | | <0.001 |
| Rural | 1.10 | 1.05 | 1.15 |  |
| **Caste** | | | | |
| Scheduled Caste | 1.20 | 1.13 | 1.28 | <0.001 |
| Scheduled Tribe | 1.13 | 1.05 | 1.22 |  |
| Other Backward Class | 1.04 | 0.99 | 1.10 |  |
| None of the casts |  | Ref | |  |
| Not sure of their caste | 1.54 | 1.22 | 1.93 |  |
| **Education** | | | | |
| No formal education | 1.58 | 1.47 | 1.69 | <0.001 |
| Completed primary education | 1.65 | 1.53 | 1.78 |  |
| Completed secondary education | 1.35 | 1.28 | 1.44 |  |
| Higher secondary and above | Ref | | |  |
| **BMI of mother** | | | | |
| Underweight | 1.61 | 1.45 | 1.78 | <0.001 |
| Normal | 1.18 | 1.08 | 1.30 |  |
| Overweight | 1.01 | 0.91 | 1.11 |  |
| Obese | Ref | | |  |
| **Wealth Quintile** | | | | |
| Poorest quintile | 1.58 | 1.48 | 1.68 | <0.001 |
| Poorer quintile | 1.43 | 1.35 | 1.53 |  |
| Middle quintile | 1.22 | 1.15 | 1.31 |  |
| Richer quintile | 1.17 | 1.08 | 1.25 |  |
| Richest quintile | Ref | | |  |
| **Pregnancy Complications** | | | | |
| No |  | Ref | |  |
| Yes | 0.99 | 0.94 | 1.04 | 0.639 |
| **Anaemia Status of mother** | | | | |
| Severe | 1.35 | 1.19 | 1.52 | <0.001 |
| Moderate | 1.07 | 1.02 | 1.12 |  |
| Mild | 1.04 | 0.99 | 1.09 |  |
| Not Anaemic | Ref | | |  |
| **Sex of the Child** | | | | |
| Female | 1.23 | 1.18 | 1.28 | <0.001 |
| Male | Ref | | |  |
| **Birth order** | | | | |
| 1^st^ child | 1.16 | 1.11 | 1.21 | <0.001 |
| 2^nd^ and 3^rd^ | Ref | | |  |
| 4^th^ and consecutive | 1.08 | 1.02 | 1.15 |  |
| **Birth Interval** | | | | |
| <24 months | 1.12 | 1.01 | 1.18 | <0.001 |
| >24 months | Ref | | |  |
| **Insurance Coverage** | | | | |
| Yes | Ref | | | <0.001 |
| No | 1.17 | 1.12 | 1.23 |  |
| **ANC Visits** | | | | |
| No visits | 1.33 | 1.22 | 1.45 | <0.001 |
| ≤4 visits | 1.12 | 1.07 | 1.17 |  |
| >4 visits | Ref | | |  |
| **Iron Supplementations** | | | | |
| No | 1.15 | 1.08 | 1.22 | <0.001 |
| Yes | Ref | | |  |
| Don’t Know | 1.45 | 0.91 | 2.33 | 0.116 |
| **Tetanus Injections** | | | | |
| No | 1.24 | 1.12 | 1.36 | <0.001 |
| Yes | Ref | | |  |
| Don’t Know | 1.55 | 1.17 | 0.21 |  |
| **Doesn’t use Cigarette and Tobacco** | | | | |
| No | 1.24 | 1.13 | 1.36 | <0.001 |
| Yes | Ref | | |  |
| **Do you drink Alcohol** | | | | |
| No | Ref | | | 0.3 |
| Yes | 1.12 | 0.91 | 1.39 |  |
| **Frequency of Alcohol Consumption** | | | | |
| Everyday | 2.55 | 1.32 | 4.89 | <0.005 |
| Once a week | 1.86 | 1.16 | 2.97 |  |
| Less than once a week | Ref | | |  |
